# Thrombotic complications of treatment with antipsychotic drugs : risk factors

**DOI:** 10.1192/j.eurpsy.2023.1234

**Published:** 2023-07-19

**Authors:** W. Abid, F. Chérif, N. Bouattour, R. Masmoudi, F. Guermazi, I. Feki, R. Sallemi, J. Masmoudi

**Affiliations:** psychiatry “A” department, hedi Chaker University Hospital, sfax, Tunisia

## Abstract

**Introduction:**

Antipsychotic agents (AP) are widely used drugs to treat psychotic symptoms. For decades, some studies suggested that there is a relationship between using (AP) and the risk of venous thromboembolism (VTE) and pulmonary embolism (PE). The causality of this association, its risk factors, and its implications for clinical practice have not been fully elucidated.

**Objectives:**

We undertook a systematic literature review to evaluate the evidence for an association between antipsychotic medication and venous thromboembolic events (VTE) and to identify risk factors for these adverse effects.

**Methods:**

To identify relevant studies, we searched the PubMed, Science Direct databases up using the following keywords « pulmonary embolism », « venous thromboembolism » « antipsychotics agents ». We also searched the reference lists relevant articles for related studies.

**Results:**

Twelve articles are included in this analysis and indicate an elevated risk of VTE in antipsychotic drug users. The results showed that compared with non-users, current AP users have significantly increased risks of VTE. The risk of venous thrombosis in obese people was higher than that in overweight people, patients not less than 65 years old had an increased risk compared with younger patients . In addition, women taking antipsychotics had a higher risk of pulmonary embolism than men. The other factors that increased risk were use of second-generation antipsychotics and antipsychotic polytherapy. The highest risk was noted in the first 3 months of treatment. Data also suggested a dose-dependent increase in the risk of thrombotic complications. For individual drugs, increased risk of VTE and PE was observed in taking clozapine , haloperidol, risperidone and olanzapine. Clozapine was associated with the highest risk. However, careful interpretation is needed because of high heterogeneity among studies and scarce data.

**Conclusions:**

The use of antipsychotics will increase the risk of venous thromboembolism and pulmonary embolism, which will be affected by AP and patient characteristics.

**Disclosure of Interest:**

None Declared

